# Glial scaffold required for cerebellar granule cell migration is dependent on dystroglycan function as a receptor for basement membrane proteins

**DOI:** 10.1186/2051-5960-1-58

**Published:** 2013-09-06

**Authors:** Huy Nguyen, Adam P Ostendorf, Jakob S Satz, Steve Westra, Susan E Ross-Barta, Kevin P Campbell, Steven A Moore

**Affiliations:** 1 Department of Pathology, 4270A Carver Biomedical Research Building, The University of Iowa, 285 Newton Road, Iowa City, IA 52242, USA; 2 Department of Molecular Physiology and Biophysics, Internal Medicine, and Neurology, The University of Iowa, Iowa City, IA 52242, USA; 3 Howard Hughes Medical Institute, Roy J. and Lucille A. Carver College of Medicine, The University of Iowa, Iowa City, IA 52242, USA

**Keywords:** Dystroglycan, Glia, Cerebellum, Development

## Abstract

**Background:**

Cobblestone lissencephaly is a severe neuronal migration disorder associated with congenital muscular dystrophies (CMD) such as Walker-Warburg syndrome, muscle-eye-brain disease, and Fukuyama-type CMD. In these severe forms of dystroglycanopathy, the muscular dystrophy and other tissue pathology is caused by mutations in genes involved in O-linked glycosylation of alpha-dystroglycan. While cerebellar dysplasia is a common feature of dystroglycanopathy, its pathogenesis has not been thoroughly investigated.

**Results:**

Here we evaluate the role of dystroglycan during cerebellar development. Brain-selective deletion of dystroglycan does not affect overall cerebellar growth, yet causes malformations associated with glia limitans disruptions and granule cell heterotopia that recapitulate phenotypes found in dystroglycanopathy patients. Cerebellar pathology in these mice is not evident until birth even though dystroglycan is lost during the second week of embryogenesis. The severity and spatial distribution of glia limitans disruption, Bergmann glia disorganization, and heterotopia exacerbate during postnatal development. Astrogliosis becomes prominent at these same sites by the time cerebellar development is complete. Interestingly, there is spatial heterogeneity in the glia limitans and granule neuron migration defects that spares the tips of lobules IV-V and VI.

**Conclusions:**

The full spectrum of developmental pathology is caused by loss of dystroglycan from Bergmann glia, as neither granule cell- nor Purkinje cell-specific deletion of dystroglycan results in similar pathology. These data illustrate the importance of dystroglycan function in radial/Bergmann glia, not neurons, for normal cerebellar histogenesis. The spatial heterogeneity of pathology suggests that the dependence on dystroglycan is not uniform.

## Background

Cerebellar histogenesis involves the formation and migration of several distinct populations of neural cells over an extended period of time, from late embryogenesis well into post-natal development [1]. This intricate and protracted developmental process renders the cerebellum susceptible to a number of insults leading to a variety of cerebellar disorders. Cobblestone lissencephaly encompasses a spectrum of brain malformations, including cerebellar dysplasia, due to defects in neuronal migration. This brain malformation is found in a clinically severe subset of congenital muscular dystrophies (CMDs)—Walker-Warburg syndrome, muscle-eye-brain disease, and Fukuyama CMD [2,3]. Genetic, pathologic, and biochemical studies in patients and in mouse models support the hypothesis that the loss of alpha dystroglycan-ligand binding underlies the muscle, eye, and brain pathology present in these disorders [4-8].

Dystroglycan (DG), a central component of the dystrophin-glycoprotein complex, is known to contribute to basement membrane structure and stability through its function as a receptor for extracellular matrix (ECM) proteins [9,10]. DG consists of an extracellular α-subunit linked to a transmembrane β-subunit. α-DG is decorated with a specific O-mannosyl glycan that functions as a high affinity receptor for ECM proteins such as laminin, agrin, and perlecan in a variety of tissues including brain [4,11,12], neurexin and Slit specifically in brain [13,14], and pikachurin in retina [15]. β-DG completes the link between the ECM and the intracellular actin cytoskeleton via its binding to α-DG outside the cell and dystrophin in the cytosol [16-19].

We previously reported that brain-selective deletion of DG in mice recapitulates some of the brain malformations characteristic of severe dystroglycanopathy, including aberrant migration of cortical and cerebellar neurons, and further showed severely blunted hippocampal long-term potentiation in these mice [5]. Subsequent studies of mice with epiblast-specific deletion of DG resulted in brain and eye malformations that broadly resemble the clinical spectrum of the human disease, including neuronal migration errors, hydrocephalus, and defects in anterior as well as posterior chambers of the eye [20]. Our most recent studies have shown DG is expressed in radial glia during neocortical histogenesis, and that glial DG is essential for multiple developmental processes, including maintenance of the basement membrane integrity, normal radial glia morphology, organization of neocortical proliferation, and cortical plate lamination [8]. Moreover, there are distinct functions for glial and neuronal DG in the cerebrum—glial DG is critical for laminar development of the forebrain, while neuronal DG is necessary for proper hippocampal longterm potentiation [7].

In the developing cerebellum, DG mRNA is detected in all major cellular populations, including radial glia, granule cell (GC) precursors, and Purkinje cells (PC) [21], while its protein expression is prominent in Bergmann glia (BG) enfeet, perivascular astrocyte endfeet, and PCs in the adult cerebellum [22]. How its expression in each of these cell types contributes to cerebellar development is not clearly understood. Here we show that DG in cerebellar GC and PC is largely dispensable for cerebellar histogenesis. In contrast, targeting both neurons and glia for DG deletion during embryonic development results in widespread, yet heterogeneous GC migration abnormalities without reducing overall cerebellar growth. Disruptions in the glia limitans and aberrant Bergmann glia (BG) organization coincide with GC migration errors and regions of gliosis, indicating that normal cerebellar histogenesis is dependent on proper BG scaffolds, which are achieved through extracellular interactions between glial α-DG and components of the ECM. These findings stress the importance of glial DG in its contribution to the basal lamina integrity during development and show that glial DG and neuronal DG have different biological functions in the cerebellum.

## Methods

### Generation of mice

**Figure 1:**
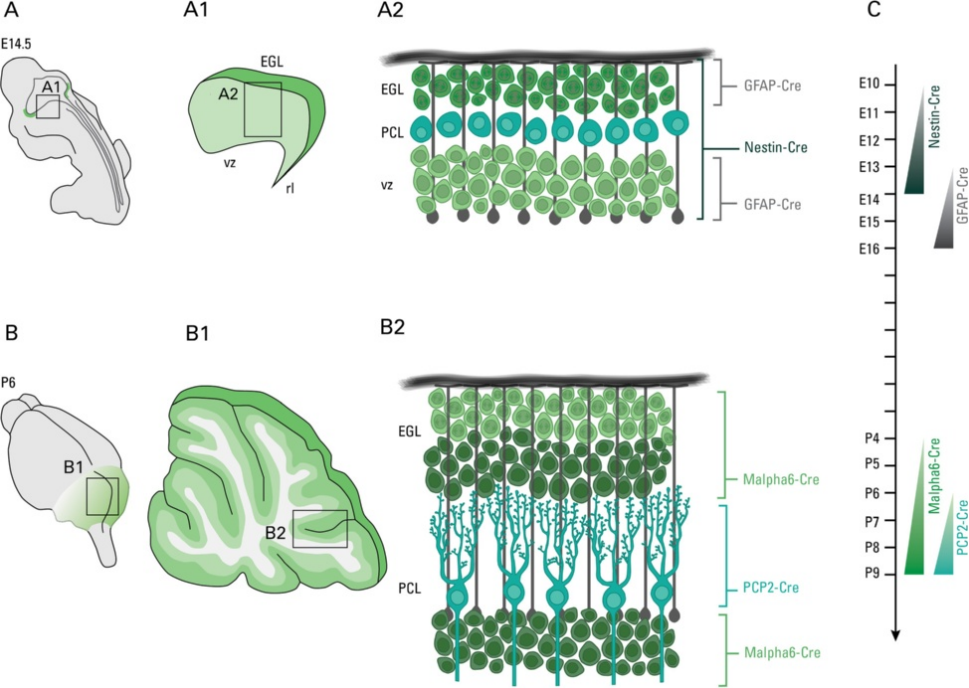
**Schematic representations of the different cellular-specific conditional dystroglycan-null mice.** Drawings illustrating the mouse cerebellum during embryonic **(A)** and postnatal development **(B)**, the cellular organization at specified developmental period **(A**1, **A**2, **B**1, **B**2**)**, and the localization of each promoter-driven Cre recombinase expression **(A**2, **B**2**)**. Timeline of Cre activities is presented in **(C)**. The nestin and GFAP promoters drive Cre expression in neuronal and glial precursors as early as E10.5 [43] and E13.5 [28], respectively. However, the GFAP promoter does not express Cre in Purkinje cells [28]. The PCP2-Cre promoter drives Cre expression in Purkinje cells beginning at P6 [23], whereas the the malpha6 promoter drives Cre expression in granule cells beginning at P4 [24].

Nestin-Cre/DG-null, PCP2-Cre/DG-null, and malpha6-Cre/DG-null mice were produced by breeding nestin-Cre mice (Jackson Labs), PCP2-Cre [23], malpha6-Cre [24] with dag1 floxed mice as described for GFAP-Cre/DG-null mice [5]. In brief, floxed DG mice (Dag1^lox/lox^) were crossed with mice heterozygous for the floxed allele and expressing Cre recombinase under the control of different promoters (Cre+/Dag1^lox/+^) to generate conditional DG-null mice (Cre+/Dag1^lox/lox^) at an expected Mendelian occurrence of 25%. Timed pregnancies were established as previously described in order to obtain embryos of specific developmental ages [8]. The cellular-specificity and developmental timing of expression for each Cre-loxP/DG-null model used in our studies are illustrated in Figure 1. Animal use procedures were approved by the University of Iowa Institutional Animal Care and Use Committee.

### Histology and immunofluorescence

For routine histology and histochemistry, postnatal mice were perfused with 4% PFA, then their brains removed and fixed for at least 24 hr by immersion in 4% PFA. Embryos and some postnatal mice were euthanized prior to fixation in Bouin’s solution. Brains were removed from older mice prior to immersion fixation, while the entire heads of embryos and younger postnatal mice were fixed in Bouin’s. Sagittal sections of the brains or heads were processed into paraffin blocks and sections cut at 6 μm. Serial sections were stained with hematoxylin and eosin and evaluated by routine light microscopy. Digital images were captured on a Zeiss microscope using standard proprietary software supplied by the manufacturers.

For immunofluorescence staining, brains were immersion fixed in 4% PFA, cryoprotected through increasing sucrose concentrations (10% to 20% to 30%) and embedded in OCT. For E14.5 embryos, the entire heads were fixed and processed for cryosections. Brains were removed from all postnatal mice prior to fixation. Alternatively, some brains were removed from postnatal mice and frozen without fixation in isopentane precooled with dry ice to approximately -75°C. Mid-sagittal sections of the cerebellum were cut on a cryostat at 10 μm, air-dried, and stored at -80°C prior to use. Stored sections were equilibrated in PBS at room temperature (RT) for 5 min and fixed for 10 min in 2% PFA. Sections were then incubated first in blocking solution (PBS containing 5% NGS, 0.1% Triton-X) for 1 hr, and then incubated with primary antibodies overnight at 4°C. Finally, sections underwent incubation with secondary antibodies for 1 hr at RT. Sections stained for BrdU were treated with 2 N HCl at 37°C for 30 min prior to blocking. All sections were counterstained with 0.01% DAPI or propidium iodide to label nuclei. Images were procured on a Zeiss ImageReady M1 fluorescence microscope. For quantification of data, ten images were obtained per section while three consecutive sections were acquired per animal, and three animals were considered per experimental group. Image adjustments and area measurements were done on Adobe Photoshop. Variabilities among data are expressed as mean ± SEM. Statistical analysis (one-way ANOVA with Tukey post-hoc test) was performed using SPSS software program and p < 0.05 was considered to be statistically significant.

### Electron microscopy

Mouse pups between P0 and P16 were perfused through the heart left ventricle with 4% PFA prior to brain removal. A 0.1 cm midsagittal slice of the cerebellum was then immersion fixed in 2.5% glutaraldehyde and further processed as an intact slice through osmication, dehydration, and embedment in epon. Selected regions of the cerebellar slices were cut out of fully polymerized epon blocks with a jeweler’s saw and glued to blank epon stubs for sectioning and ultrastructural evaluation.

### BrdU labeling

Mice received intraperitoneal injections of bromodeoxyuridine (BrdU, 50 mg/kg) 30 min prior to euthanasia to assess external granule cell proliferation or several days prior to euthanasia to assess granule cell migration.

### Antibodies

The following primary antibodies are used: IIH6 (anti-αDG [9]), AP83 (anti-βDG [25]), anti-β1Itg [26], anti-AQP4 (Millipore AB3068), anti-BrdU (Abcam ab6326), anti-BLBP (Millipore AB9558), anti-calbindin (Millipore AB1778), anti-collagen IV (Abcam ab19808), anti-GABARA6 (Sigma G5544), anti-GFAP (Sigma G3893), anti-laminin (Sigma L9393), anti-Kir4.1 (Alomone Labs APC-035), anti-MAP2 (Sigma M4403), anti-NeuN (Millipore MAB377), anti-pax6 [Developmental Studies Hybridoma Bank (DSHB) PAX6] anti-perlecan (Millipore MAB1948), anti-shh (Abcam ab50515), anti-tag1 (DSHB 4D7), and anti-nestin (DSHB rat-401).

Secondary antibodies used in all experiments are Alexa Fluor fluorescent dye conjugates (Invitrogen): goat anti-mouse IgG1, goat anti-mouse IgM, goat anti-rabbit IgG, and goat anti-rat IgG.

## Results

### Neuronal dystroglycan is not essential for cerebellar histogenesis

**Figure 2:**
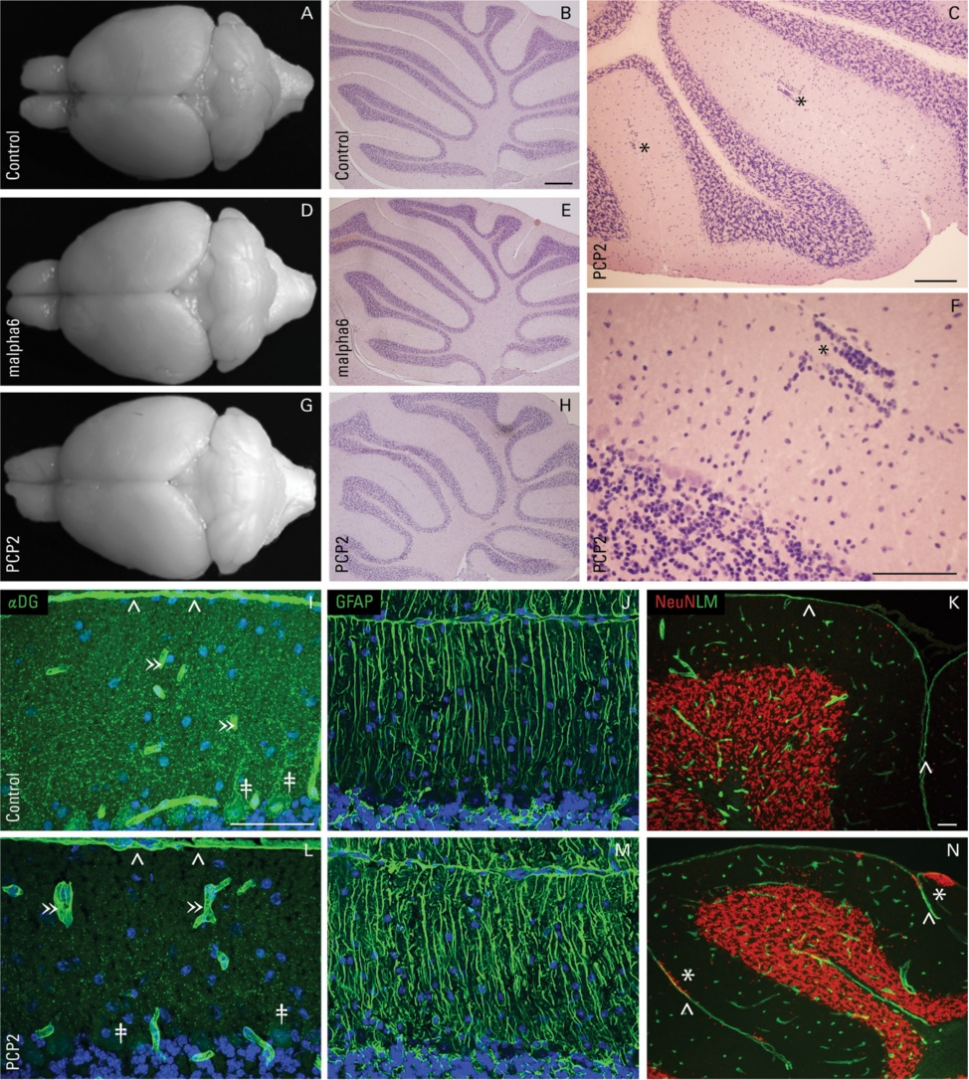
**Development in cerebellar neuron conditional dystroglycan-null mice.** Macroscopic images of cerebellar morphology and hematoxylin-eosin stained, mid-sagittal sections of adult cerebella from control **(A**, **B)**, malpha6-Cre/DG-null **(D**, **E)**, and PCP2-Cre/DG-null **(G**, **H)** mice. Higher magnification of PCP2-Cre/DG-null mid-sagittal cerebellar section showing small heterotopia **(C**, **F)**. Immunofluorescent staining of α-dystroglycan (αDG) and glial fibrillary acidic protein (GFAP) (both green) in control **(I**, **J)** and PCP2-Cre/DG-null cerebella **(L**, **M)**. Double staining of laminin (LM; green) and neuronal nuclear protein (NeuN; red) in control **(K)** and PCP2-Cre/DG-null **(N)** cerebella. The cerebellum of both malpha6-Cre/ and PCP2-Cre/DG-null develop normally without gross malformations. Dystroglycan is expressed as puncta in Purkinje cells (double daggers; ‡) and concentrated at glial endfeet (carets; ^) as well as blood vessels (right double angle brackets; ») in adult control cerebellum **(I)**. In the PCP2-Cre/DG-null mice, dystroglycan expression is greatly reduced in Purkinje cells, but its expression in glial cells remains unaltered at the glia limitans and surrounding vessels **(L)**. Loss of dystroglycan expression in the PCP2-Cre/DG-null does not affect Bergmann glia morphology **(M)** while causes in a mild degree of GC ectopia (asterisks; *) immediately beneath an intact glia limitans (carets; ^) **(N)**. DAPI (blue) was used as a nuclear counter stain. Scale bar: 20 μm.

Dystrolygcan (DG) is broadly expressed in most cellular populations of the developing cerebellum—pre-migratory granule cells, Purkinje cells, and radial/Bergmann glia [21]. To evaluate how DG expression in each of the aforementioned cell types contributes to cerebellar histogenesis, we generated cell-specific conditional DG-null mice (Figure 1). We first examined the cerebellum of PCP2-Cre/DG-null mice, where DG is conditionally deleted only in PCs, then in malpha6-Cre/DG-null mice, where DG is conditionally deleted from GCs (Figure 2).

Dystroglycan is expressed in Purkinje cells (PCs) during early postnatal cerebellar development [21], and DG-positive puncta rimmed the PC somata and decorated PC dendrites across the molecular layer in the adult (P21) control cerebellum (Figure 2I). To assess whether loss of DG in PC is adequate to cause migration defects, we evaluated the histopathology of PCP2-Cre/DG-null mice. The PCP2 promoter drives Cre expression in PCs beginning on postnatal day 6 [23]. The brains of these mice were grossly normal and many histologic sections were free of histopathology (Figure 2G, H). After thorough examination of several adult mice (n = 9), small, infrequent GC heterotopia were identified in the molecular layer of each cerebellum (Figure 2C, F), just internal to an intact glia limitans (Figure 2N). No disruptions of the glia limitans or abnormalities of Bergmann glia orientation were observed (Figure 2M, N) despite a nearly complete loss of PC dystroglycan (Figure 2L).

Granule cells in the cerebellar cortex express DG during their migration and lose expression during maturation in the internal granule cell layer, suggesting that expression of DG in GC may play a role during their migration [21]. To determine whether the loss of DG in GC is sufficient to cause defects in GC migration, the histopathology of the cerebellum was examined in malpha6-Cre/DG-null mice, where DG is conditionally deleted from GCs. The malpha6 promoter drives Cre expression in GCs beginning at postnatal day 4 [24]. The brains of these animals (n = 7) were grossly normal (Figure 2D) and GC ectopia were not observed (Figure 2E).

### Cerebellar growth in conditional dystroglycan-null mice

**Figure 3:**
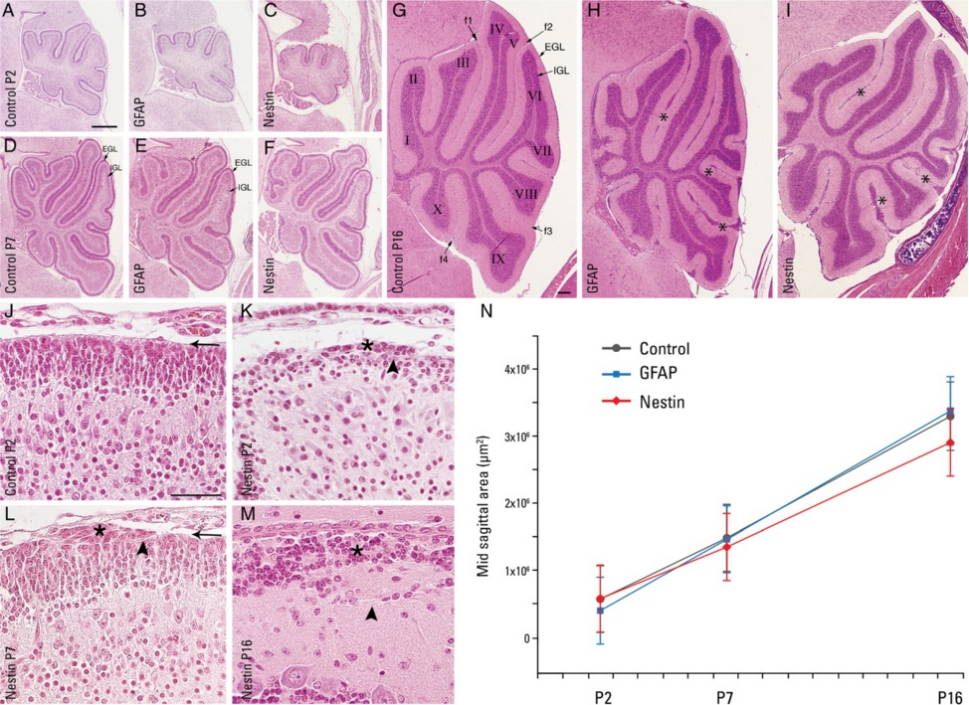
**Cerebellar growth in brain-selective conditional dystroglycan-null mice.** Cerebellar growth and development from P2, P7, and P16 in control **(A**, **D**, **G)**, GFAP-Cre/DG-null **(B**, **E**, **H)**, and nestin-Cre/DG-null **(C**, **F**, **I)**. Normal lobules and fissures are indicated in the P16 control cerebellum **(G)**. In both nestin-Cre/DG-null and GFAP-Cre/DG-null mice, many lobule surfaces and fissures display abnormal numbers of ectopic cells (asterisks; *) on the cerebellar surface or between two adjacent lobules. The resulting pathology appears as fused fissures and heterotopic neurons. Basement membrane (arrows; ➛) at the glia limitans is continuous along the surface of controls **(J)**. In nestin-Cre/DG-null cerebellum at P2 **(K)**, a cluster of granule neurons (asterisk; *) is seen outside the glia limitans (arrowhead; ➤). Similar, but more extensive, pathology is seen at P7 **(L)** and P16 **(M)**. Area measurements from mid-sagittal sections of control, GFAP-Cre/DG-null, and nestin-Cre/DG-null at P2, P7, and P16 **(N)**. Error bars denote standard error of the mean. n = 3 for each group. EGL = external granule cell; IGL = internal granule cell layer; Scale bar: 40 μm **(A**-**I)**, 40 μm **(J**-**M)**.

After establishing that neuronal DG is not necessary for cerebellar histogenesis, we next evaluated the role of glial DG in cerebellar development by using mice with deletion of DG from both neuronal and glial cells. The generation of the GFAP-Cre/DG-null and nestin-Cre/DG-null mice has been previously described [5,7,8]. The nestin promoter drives Cre expression in neuronal and glial precursors as early as embryonic day 10.5, while the ependyma and choroid plexus show no expression of the nestin-Cre transgene [27]. The GFAP promoter drives expression of Cre recombinase in radial glia, astrocytes, ependyma and some neuronal cell types beginning at embryonic day 13.5, but Purkinje cells and the choroid plexus do not express GFAP-Cre [28]. The cerebella of these mice were grossly normal; area measurements of mid-sagittal sections from three different post-natal developmental time points showed no significant differences among the two mutant genotypes and their littermate controls (Figure 3N). However, several ectopic foci of granule cells were noted in the cerebella of both GFAP-Cre and nestin-Cre conditional DG-null mice at post-natal day 8 (Additional file 1: Figure S1) and day 16 (Figure 3H, I). Under higher magnification, these ectopic cells appeared as clusters of non-migrating GCs that remained in the EGL along some fissures and at the surfaces of some lobules, with at least some granule neuron clusters migrating outward, through gaps in the basal lamina, into the leptomeninges (Figure 3, K-M). By P21 this pattern of dysgenesis appeared as fused interlobular fissures (Additional file 2: Figure S2). PCs in the meanwhile developed normally albeit for focal secondary irregularities in dendritic arborization—some dendrites reached across adjacent lobules at areas of discontinuous basal lamina and GC ectopia (Additional file 3: Figure S3).

### Dystroglycan expression in the developing cerebellum

**Figure 4:**
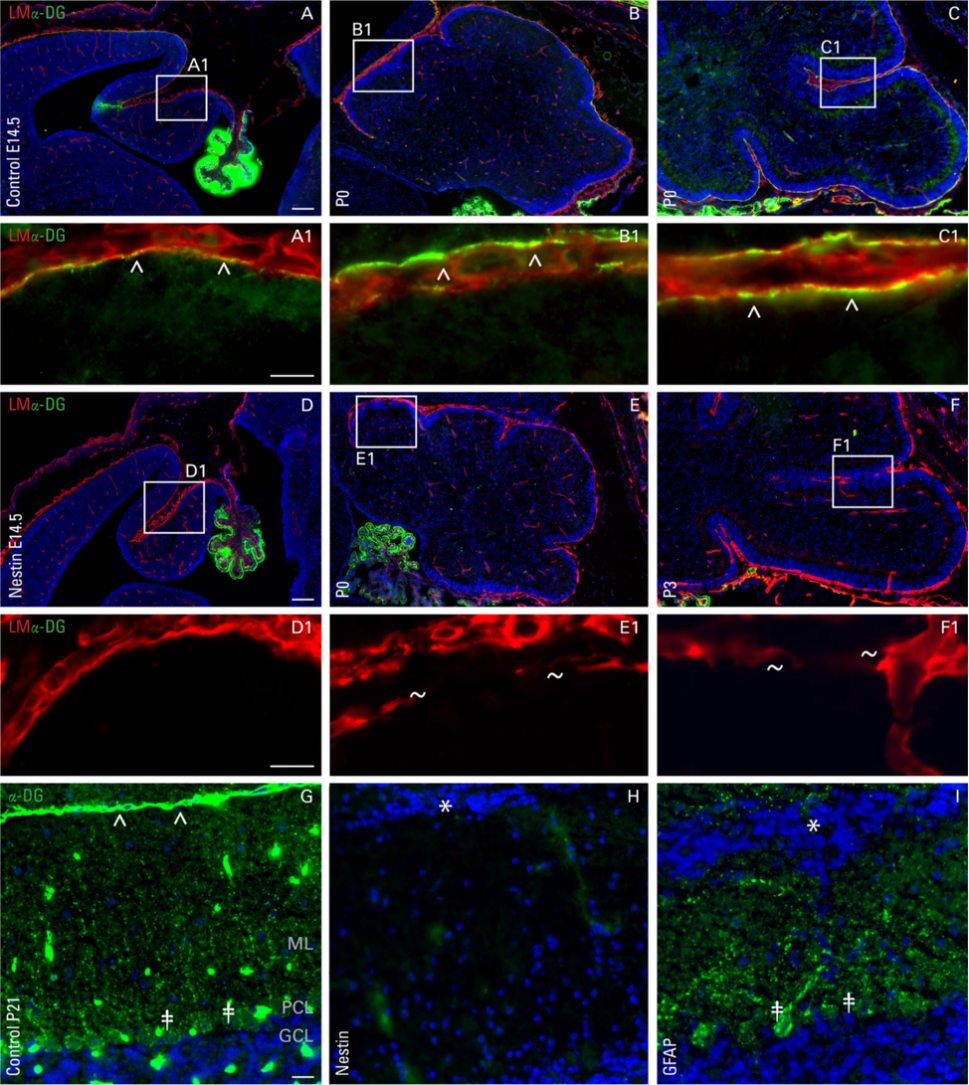
**Expression of dystroglycan in the developing cerebellum.** Immunofluorescent detection of α-dystroglycan (green; α-DG) and laminin (red; LM) in the cerebellum of wild-type littermate controls at E14.5, P0, P3, and P21 **(A**-**C**, **G)**, nestin-Cre/DG-null mice at matching developmental ages **(D**-**F**, **H)**, and GFAP-Cre/DG-null at P21 **(I)**. DG is detectable at E14.5 and localizes with laminin (carets; ^) at the basement membrane overlying the cerebellar primordium. In the absence of dystroglycan at E14.5 in the nestin-Cre/DG-null, the basement membrane appears to be intact, while small discontinuities are noticeable at P0 and exacerbates by P3 (tildes). DG expression in Purkinje cells (double daggers; ‡) is retained in the GFAP-Cre/DG-null, but not the nestin-Cre/DG-null cerebellum. DAPI (blue) was used as a nuclear counter stain. Asterisk (*) denotes ectopic cells. GCL = granule cell layer; ML = molecular layer; PCL = Purkinje cell layer. Scale bar: 20 μm.

To evaluate DG expression in the developing cerebellum, mid sagittal sections from cerebella of nestin-Cre/DG-null and littermate controls were double stained for α-DG and laminin, a basement membrane protein and ligand of αDG. DG was found to be concentrated at the choroid plexus and the glia limitans from at least embryonic day 14 (E14.5), and it colocalized with laminin at the glia limitans (Figure 4, A-C). Loss of DG in the nestin-Cre/DG-null cerebellar anlage was confirmed at E14.5 (Figure 4D). Laminin staining remained indistinguishable from control tissue, indicating an intact basement membrane at this developmental time point. Soon after birth (P0), laminin expression at the basal lamina was absent in small foci (Figure 4E), but the extent of abnormal laminin immunostaining spread as the cerebellum grew to P3 (Figure 4F). These breaks at the basal lamina were found at the tips of cerebellar lobules and along fissures between lobules of the nestin-Cre/DG-null mice. In the developmentally mature control cerebellum (P21), DG was expressed in Purkinje cells (PCs) in addition to being concentrated at glial endfeet (Figure 4G). Both neuronal and glial DG were lost in the nestin-Cre/DG-null mice (Figure 4H), whereas the GFAP-Cre/DG-null mice showed loss of DG from glial cell endfeet, while maintaining expression in PCs (Figure 4I).

### Disruptions at the glia limiting membrane correlate with radial glia/Bergmann glia irregularities

**Figure 5:**
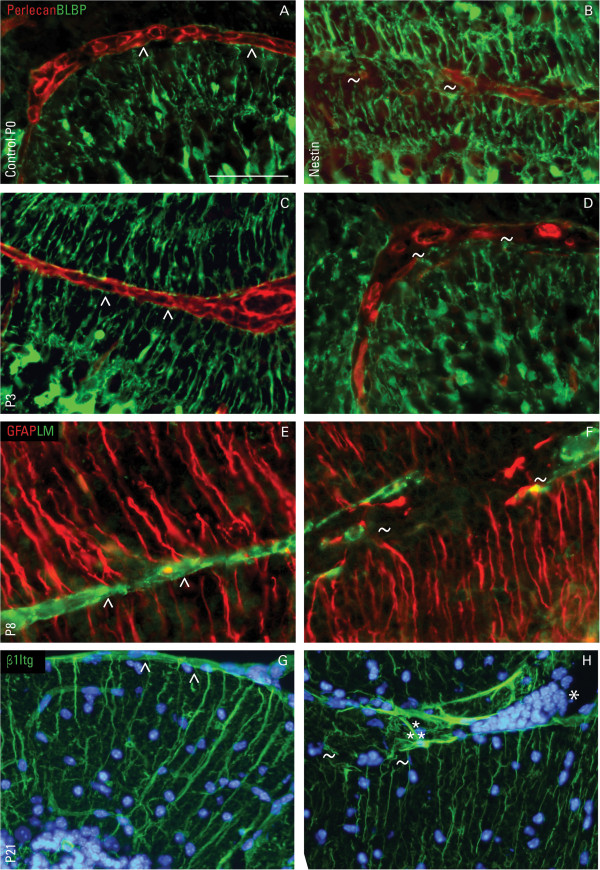
**Focal disruptions at the basement membrane are correlated with disorganized radial glia.** Immunofluorescent detection of perlecan (red) and brain-lipid binding protein (BLBP; green) in control **(A**, **C)** and nestin-Cre/DG-null cerebella **(B**, **D)** on the day of birth (P0) and at P3. Immunofluorescent staining of glial fibrillary acidic protein (GFAP; red) and laminin (LM; green) in control **(E)** and nestin-Cre/DG-null littermate **(F)** at P8. Immunostaining of β1-integrin (β1Itg, green) in control **(G)** and nestin-Cre/DG-null cerebella **(H)** at P21. In control cerebella, radial glia (Bergmann glia at P8) endfeet abut an intact basement membrane (carets), forming the continuous glia limitans. In the absence of DG, disruptions at the basement membrane are often found in areas of disorganized radial glia throughout development (tildes; ~). β1Itg is normally expressed in Bergmann glia in both control and nestin-Cre/DG-null cerebella. However, β1Itg expression appears to be upregulated at areas of gliosis (asterism; ⁂), along with ectopic cells (asterisks; *) and disrupted glia limitans (tildes; ~). DAPI (blue) was used as nuclear counter stain. Scale bar: 20 μm.

**Figure 6:**
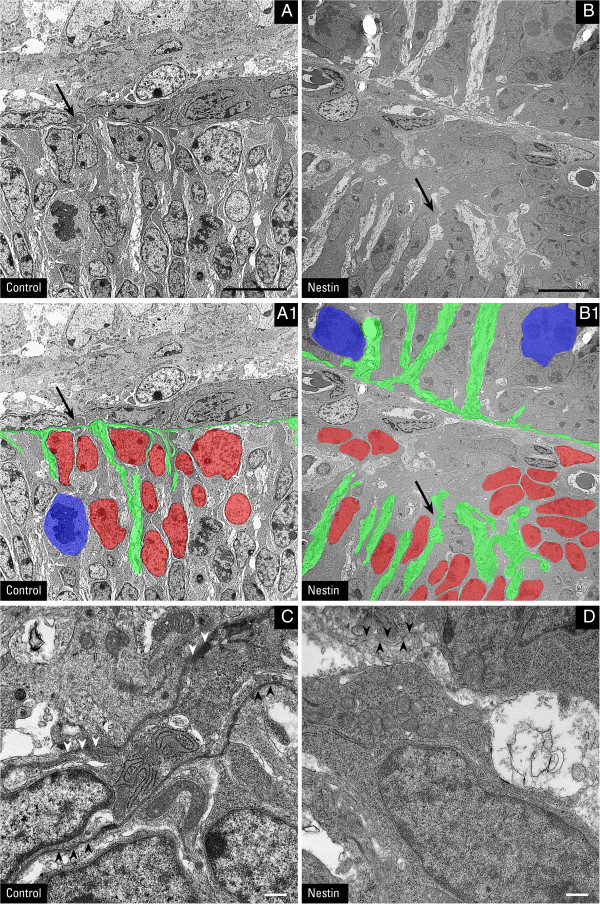
**Electron micrographs of breaks in the glia limitans.** Electron micrographs are from nestin-Cre/DG-null cerebellum and littermate control at P7. In panels **A**1 and **B**1 **(**duplicates of panels **A** and **B)**, a subset of Bergmann glia processes are shaded in green while selected GCs are colored in red and GCs undergoing mitosis are painted in blue. Panels **C** and **D** are higher magnification images of the regions of interest indicated by arrows in panels **A**, **A**1, **B**, and **B**1. A continuous basal lamina (arrowheads) lines either side of this fissure in a control P7 cerebellum **(C)**. A DG-null littermate **(D)** displays a region containing abnormally organized radial glia processes and a largely absent basal lamina. Cells have migrated into the fissure, leaving a fragment of the glia limitans (arrowheads) buried beneath heterotopic cells. Scale bar: 10 μm **(A** and **B)**; 0.5 μm **(C** and **D)**.

**Figure 7:**
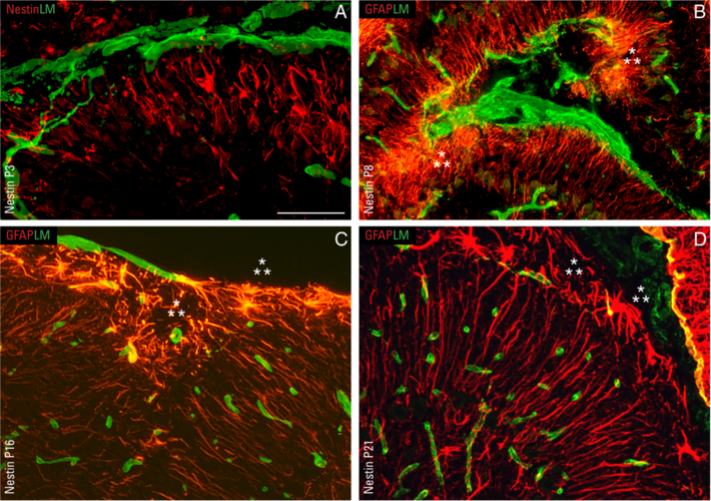
**Disruptions in the glia limitans are associated with reactive astrocytes.** Immunofluorescent detection of nestin (red) and laminin (LM; green) in nestin-Cre/DG-null cerebellum at P3 **(A)**. Immunofluorescent staining of glial fibrillary acidic protein (GFAP; red) and laminin (LM; green) in nestin-Cre/DG-null cerebella at P8 **(B)**, P16 **(C)**, and P21 **(D)**. Reactive astrocytes (asterisms; ⁂) dominate areas where the basement membrane, demarcated by laminin staining, is severely disrupted or mostly absent. Scale bar: 20 μm.

Since DG is normally expressed and concentrated at glial endfeet abutting the glia limitans, we next assessed the morphology and orientation of radial glia (Bergmann glia) in the absence of DG. Brain lipid binding protein (BLBP) and nestin were used as markers for radial glia at P0 and P3 due to their expression early in cerebellar development, whereas glial fibrillary acidic protein (GFAP) was employed as a glial marker from P8 onward. Perlecan and laminin are protein constituents of the extracellular matrix that are ligands for α-DG. Radial glia (and later Bergmann glia) are oriented in such a way that their endfeet abut and form the glia limiting membrane underneath the basement membrane (Figure 5A, C). In the nestin-Cre/DG-null cerebellum, small breaks at the glia limitans that were first observed at P0 then became much more visible at P3, and were accompanied by projections of glial processes into these gaps (Figure 5B, D). Breaches became exacerbated as the basement membrane, visualized by both DG ligands (laminin and perlecan) and a non-DG ligand (type IV collagen; Additional file 4: Figure S4), became severely disrupted by P8, while Bergmann glial endfeet projected into the gaps at the basement membrane or retracted underneath the EGL (Figure 5F). Electron microscopy studies confirmed the presence of disrupted basement membrane, abnormally organized glial endfeet, and ectopic cells within the leptomeninges of nestin-Cre/DG-null cerebella (Figure 6). Additionally, β1 integrin (β1itg), a laminin receptor, was expressed in Bergmann glia and concentrated at glial endfeet (Figure 5G); a compensatory change in β1itg expression was not observed at the glia limitans in the nestin-Cre/DG-null mice, although focal areas of gliosis did show high levels of expression (Figure 5H). Furthermore, we observed reactive gliosis at sites with disrupted basement membrane in developmentally more mature mice (Figure 7). Taken together, our data indicate that the expression of DG in glial cells is important for their morphological development as well as the integrity of the surface basement membrane during cerebellar growth.

We further evaluated the expression of proteins known to associate with DG and the dystrophin-glycoprotein complex (DGC) in glial cells, namely aquaporin 4 (AQP4) and potassium inward rectifying channel 4.1 (Kir4.1). The membrane pore protein AQP4 comprising the major water channels in brain is clustered together with the DGC via interaction between AQP4 and α-syntrophin [29], which in turn binds to dystrophin and β-DG. In the cerebellum, AQP4 concentrated at the glia limitans in control (Additional file 5: Figure S5A, B) but was reduced at P8 and lost by P16 in the nestin-Cre/DG-null mice (Additional file 5: Figure S5D, E). Whether loss of AQP4 at glial endfeet in these mice causes other pathologic phenotypes remains to be investigated, but AQP4-null mice show no brain morphological defects, suggesting that the presence of ectopic cells in the nestin-Cre/DG-null mice is independent from AQP4. Kir4.1 is enriched at Müller glial endfeet abutting the inner limiting membrane in the retina; defects in Kir4.1 clustering and retinal physiology have been demonstrated in the nestin-Cre/DG-null mice [6]. In the cerebellum, Kir4.1 is expressed in the molecular layer, but is not concentrated at the glia limitans. Expression of Kir4.1 was indistinguishable between nestin-Cre/DG-null and littermate control (Additional file 5: Figure S5C, F), indicating that Kir4.1 localization in the cerebellum, unlike in the retina, is not dependent on DG.

### Proliferation and migration of postnatal granule cells

**Figure 8:**
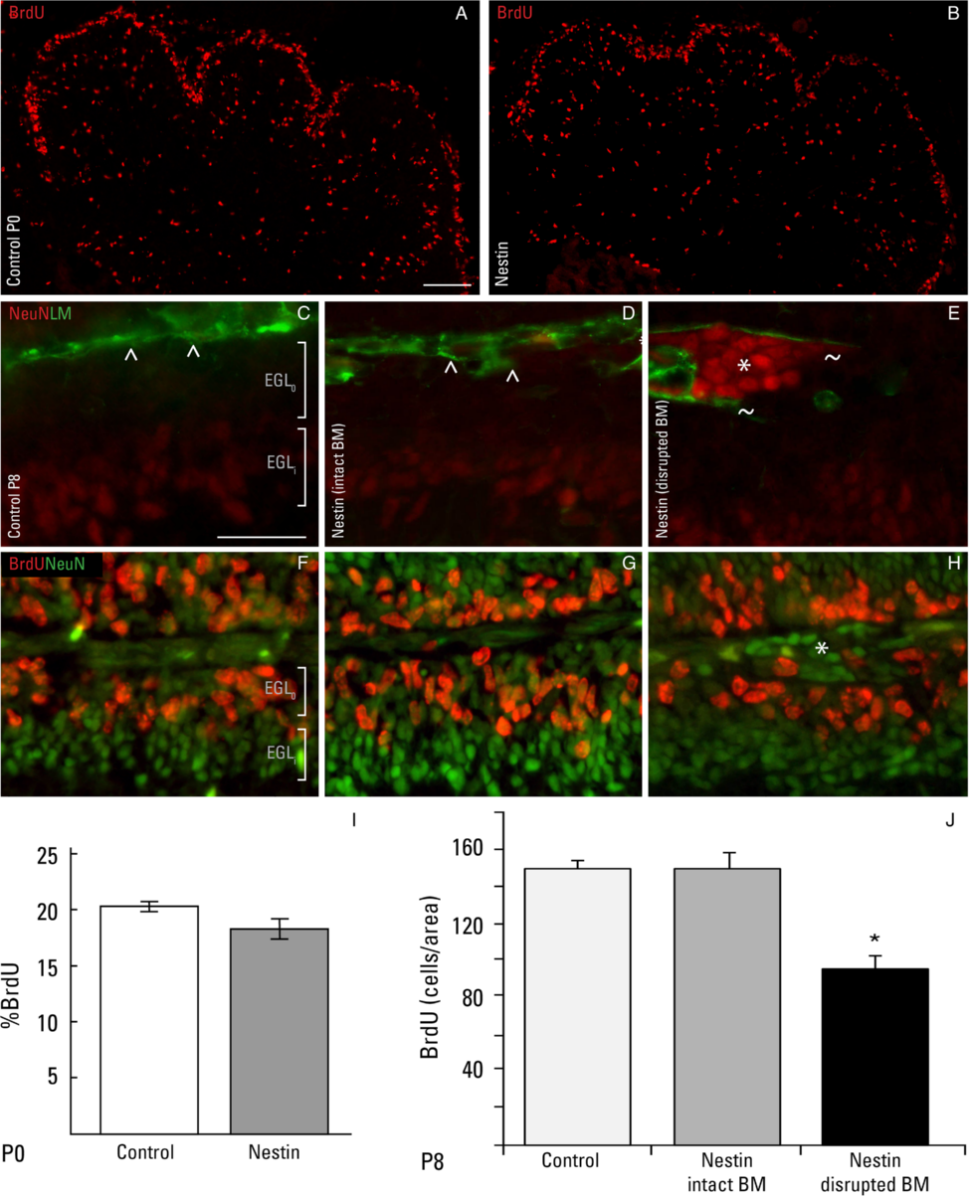
**Proliferation of granule cells.** Immunofluorescent detection of 5-bromodeoxyuridine (BrdU; red) in cerebella of control **(A)** and nestin-Cre/DG-null mice **(B)** at P0. Immunofluorescent detection of neuronal nuclear protein (NeuN; red) and laminin (LM; green) in cerebella of control **(C)** and nestin-Cre/DG-null mice **(D**, **E)** at P8. Immunofluorescent detection of 5-bromodeoxyuridine (BrdU; red) and neuronal nuclear protein (NeuN; green) in control **(F)** and nestin-Cre/DG-null cerebella at P8 **(G**, **H)**. Quantitative analysis of GC proliferation at P0 **(I)** and P8 **(J)**. GC proliferation appears to be normal in control and nestin-Cre/DG-null cerebella at birth. However, a reduction in the number of BrdU positive cells was detected at areas of disrupted basement membrane in the nestin-Cre/DG-null cerebellum at P8. Carets (^) denote the intact basement membrane; tildes (~) indicate areas of disrupted basement membrane; asterisks (*) represent ectopic GCs. Error bars denote standard error of the mean. Asterisk in panel J denotes P < 0.05. n = 3 for each group described in bar graphs. b.m. = basement membrane; EGL_i_ = inner external granule cell layer; EGL_o_ = outer external granule cell layer. Scale bar: 20 μm.

To assess GC proliferation immediately after birth (P0) and again at the peak of cerebellar growth (P8), 5-bromodeoxyuridine (BrdU) was acutely given to mice 30 minutes prior to sacrifice, and cerebellar tissues were evaluated in cryosections using an antibody against BrdU. BrdU strongly labeled the EGL at P0 and the outer proliferative EGL (EGL_o_) at P8 in the control cerebellum (Figure 8A, F). The nestin-Cre/DG-null mice at P0 showed a similar expression profile of BrdU positive cells and were not significantly different from the control tissues (Figure 8B, I). At P8, ectopic GCs labeled by neuronal nuclei protein (NeuN) and residing in areas of highly disruptive basal lamina (Figure 8E, H) have stopped proliferating. Quantitative analysis showed a significantly reduced number of BrdU-positive cells in regions with a disrupted basal lamina (Figure 8J) compared to cells beneath an intact basement membrane (Figure 8G) and compared to control cerebellum (Figure 8F).

**Figure 9:**
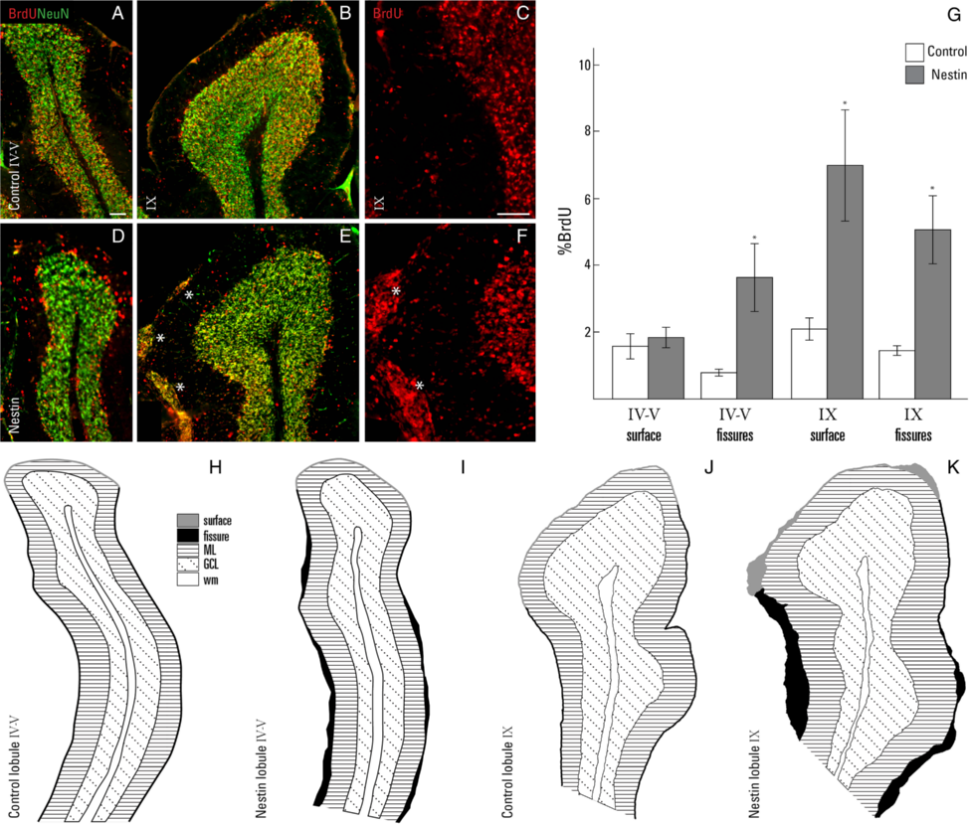
**Migration of granule cells.** Immunofluorescent detection of 5-bromodyoxyuridine (BrdU; red) and neuronal nuclear protein (NeuN; green) in cerebella of control **(A**-**C)** and nestin-Cre/DG-null **(D**-**F)** mice at P16. Quantitative analysis of GC migration from P8 to P16 **(G)**. Representative schematic of lobules IV-V and IX from a control **(H**, **J)** and nestin-Cre/DG-null **(I**, **K)**. GC from the EGL proliferate, differentiate and migrate inward to form the GCL. In the nestin-Cre/DG-null mice, some of these cells undergo differentiation but remain at the cerebellar surface or locate within fissures instead of migrating to their proper destination in the IGL. The surface of lobule IV-V is not affected in the nestin-Cre/DG-null cerebellum. Asterisks (*) indicate ectopic GCs **(D**-**F)**. Error bars denote standard error of the mean. Asterisks in the graph denote P < 0.05. n = 3 for each group described in bar graphs. GC = granule cell; GCL = granule cell layer; IGL = internal granule cell layer. Scale bar: 20 μm.

To investigate the fate of GC produced during the latter half of cerebellar histogenesis, pulse-chase migration studies were performed by BrdU administration at P8 and evaluation of cerebellar tissues at P16. In the control mice, the vast majority of cells labeled at P8 were found within the IGL at P16. In contrast, many of these later-born GCs remained at the cerebellar surface within ectopia of the nestin-Cre/DG-null cerebellum (Figure 9).

We additionally evaluated expression levels of sonic hedgehog (Shh)—a critical morphogen involved in the patterning of many brain structures during development—at P8 and P16 to confirm that GC proliferation and migration defects were independent from Shh pathways. Shh expression in the cerebellum was found to be comparable between nestin-Cre/DG-null and control littermates (Additional file 4: Figure S4).

**Figure 10:**
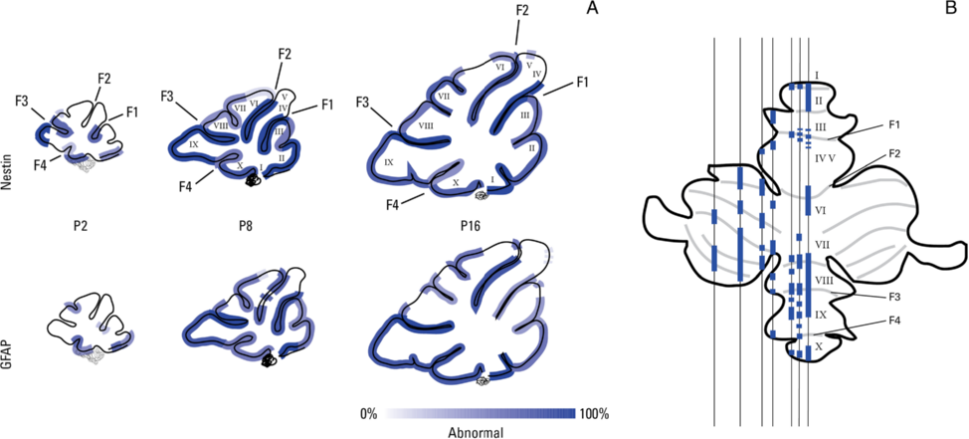
**Neuronal migration errors are not spatially uniform.** GC migration errors discerned from H&E-stained paraffin sections of nestin-Cre/DG-null and GFAP-Cre/DG-null cerebella (see Figure 3 and Additional file 6: Table S1) were plotted in mid-sagittal maps **(A)**. Darker shading reflects a higher proportion of samples displaying pathology in a specific region of the cerebellum. Nestin-Cre/DG-null cerebella display more widespread clusters of ectopic GC. The surface of lobules IV-V, and an area on the surface of lobule VI, are relatively spared. A surface map of GC migration errors from a representative nestin-Cre/DG-null cerebellum at P8 is illustrated **(B)**. Pathologic clusters of non-migrating GC exist over a vast area of lobule surfaces and fissures at every parasagittal level examined.

In comparing the degree of glia limitans pathology and ectopic GC over entire mid-sagittal sections, sparing of lobules IV-V and VI was noted (Additional file 6: Table S1). A quantitative comparison of lobules IV-V and IX was carried out in nestin-Cre/DG-null mice (Figure 9G), and complete mapping of mid-sagittal sections was performed in both nestin-Cre/DG-null and GFAP-Cre/GFAP-null mice (Figure 10A). The spatially asymmetric pathology had the greatest degree of sparing limited to the tips of lobules IV-V; the tip of lobule VI was spared to a lesser degree. Fissures between these lobules were not spared in comparison to any other fissures. This analysis also showed gradually more severe pathology over an increasingly larger surface area of the developing cerebellar cortex in both nestin-Cre/ and GFAP-Cre/DG-null mice, with the former showing a somewhat more severe phenotype at each postnatal age evaluated. Evaluation of parasagittal sections showed that glia limitans and GC pathology extended laterally to involve the cerebellar hemispheres in both models. The spatial distribution of pathology away from the midline in one nestin-Cre/DG-null cerebellum was mapped (Figure 10B).

### Characterization of ectopic granule cells at sites of disrupted basal lamina

**Figure 11:**
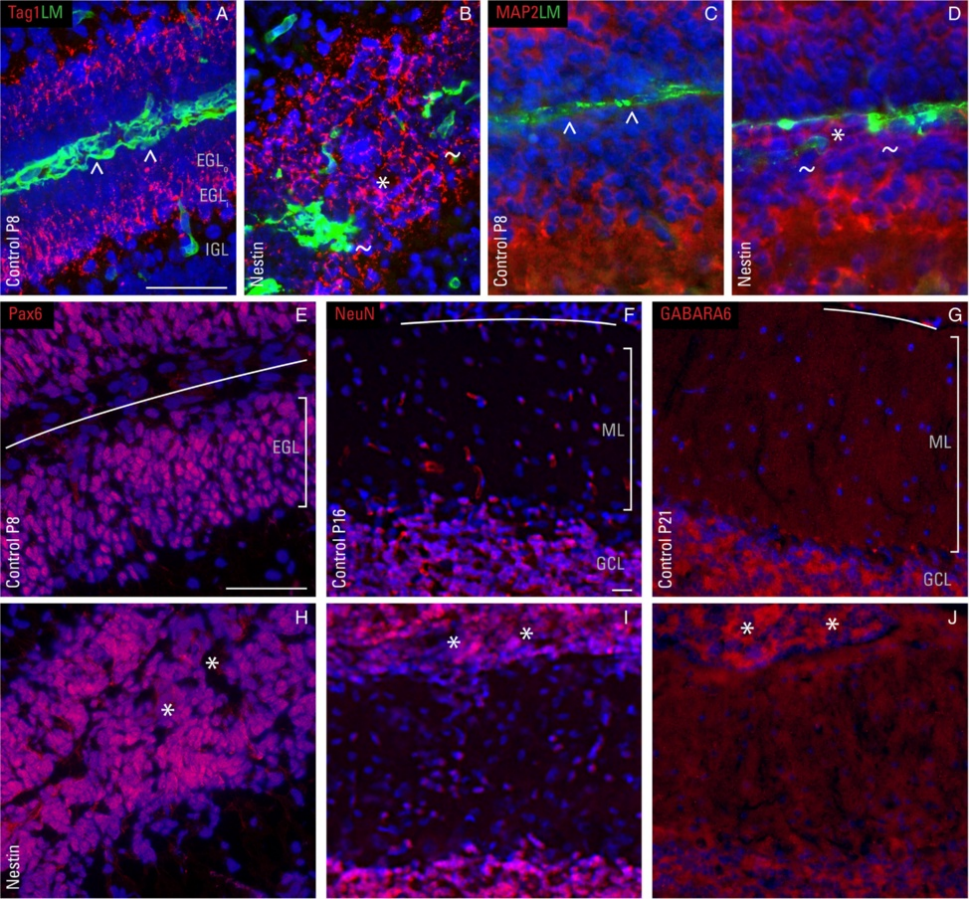
**Ectopic neurons are present at areas of disrupted basement membrane.** Immunofluorescent detection of transient axonal glycoprotein 1 (TAG1), microtubule-associated protein 2 (MAP2), paired box 6 (pax6), neuronal nuclear protein (NeuN), and GABA_A_ receptor α6 (GABARA6) (all red) and laminin (LM; green) in control cerebella at P8 **(A**, **C**, **E)**, P16 **(F)**, P21 **(G)** and in age-matched nestin-Cre/DG-null **(B**, **D**, **H**-**J)**. At P8, the EGL in the control cerebellum consists of an outer and inner layer, corresponding to proliferating and post-mitotic GC, respectively. Toward the end of cerebellar development at P16, the EGL ceases to exist as differented GCs migrate inward to form the IGL. In the absence of dystroglycan, ectopic GC differentiate yet remain trapped at the cerebellar surface where the basement membrane is highly disrupted. DAPI (blue) was used as a nuclear counter stain. Carets (^) denote the continuous basement membrane; tildes (~) indicate areas of disrupted basement membrane; asterisks (*) represent ectopic cells. Solid lines in panels E-G demarcate the continuous basal lamina. EGL_i_ = inner external granule cell layer; EGL_o_ = outer external granule cell layer. GCL = granule cell layer; IGL = internal granule cell layer; ML = molecular layer. Scale bar: 20 μm.

To assess GC maturation, the expression of several markers was evaluated in the course of post-natal cerebellar development. The inner, post-mitotic, pre-migratory zone of the EGL (EGL_i_) was labeled with transient axonal glycoprotein 1 (tag1) and microtubule-associated protein 2 (MAP2) at P8 (Figure 11A, C). Tag1 is down-regulated as GCs begin to migrate inward across the molecular layer (ML) to settle in the internal granule cell layer (IGL); tag1-null mice displayed a mild GC migratory defect [30]. MAP2 is a cytoskeletal protein that plays essential roles in a number of cellular developmental processes, including neuronal morphogenesis. In the nestin-Cre/DG-null cerebellum, tag1-positive cells were visible within the EGL_o_ as ectopia bridging between adjacent lobules (Figure 11B); MAP2 showed similar expression to tag1 in small foci of ectopic GCs (Figure 11D). Meanwhile, the paired box 6 (pax6) transcription factor, a DNA-binding protein crucial for CNS development, was shown in cells of the EGL underneath an intact basement membrane (Figure 11E). GCs from Pax6-null mice, due to defect in cytoskeletal organization and polarization, do not form leading edges and migrate in random directions instead of radially inward [31]. The expression level of pax6 in ectopic cells, at sites of disrupted basement membrane, was similar to that of surrounding cells and in control (Figure 11H). These data indicate that the cellular machinery responsible for GC maturation is largely unperturbed in the nestin-Cre/DG-null cerebellum, insofar as we have demonstrated, and that the cause of ectopia is seemingly due to aberrant glial organization and reactive gliosis rather than GC-intrinsic abnormality. In support of this interpretation, GC ectopia at later developmental stages (P16 and P21) expressed markers of mature neurons normally found in the granule cell layer (GCL), for instance NeuN and GABA_A_ receptor α6 (GABARA6) (Figure 11I, J).

## Discussion

In addition to dystrophic skeletal muscle pathology, a spectrum of developmental brain abnormalities are associated with congenital muscular dystrophies caused by mutations in genes involved in the glycosylation pathways of dystroglycan [32,33]. Cysts, hypoplasia, or dysplasia are among the prominent cerebellar pathologic features frequently observed in these dystroglycanopathy patients [2,3]. Dystroglycan is expressed in important cell types of the cerebellum, including Bergmann glia, GCs, and PC [21,22]. In this study, we show that glial DG, via interaction with the ECM at the glia limitans, is critical for normal cerebellar histogenesis, while neuronal DG is largely unnecessary.

The predominant phenotype observed in our conditional DG knockout models of cerebellar dysplasia is widespread GC migration errors, corroborated by BrdU pulse-chase labeling; a subset of GCs expressing markers of mature neurons remain on the EGL surface, at sites of disrupted glia limitans and aberrant glial organization as well as reactive gliosis. These phenotypes are remarkably similar to those described in previous cerebellar development studies of β1Itg-null mice [26]. β1Itg is another laminin receptor that plays a role in GC precursor proliferation via its interaction with sonic hedgehog (Shh) and laminin. In the absence of β1Itg, GCs lose contact with the basement membrane, exit the cell cycle, and differentiate prematurely [26,34]. Similar to β1Itg-null mice, our DG-null mice showed reduced GC proliferation at areas of highly disrupted glia limitans. However, further investigation showed relatively normal Shh expression in the absence of DG, and that Shh is even present in areas of ectopic GC (Additional file 4: Figure S4). Additionally, β1Itg itself is unable to compensate for the loss of DG, as its expression in Bergmann glia is not affected in the nestin-Cre/DG-null cerebellum (Figure 5H). Moreover, CNS deletion of integrin-linked kinase, an intracellular effector of β1Itg, results in abnormal cerebellar development associated with defects in glial process outgrowth, meningeal basement membrane assembly, and GC proliferation [35,36]. In contrast, deletion of the cytoplasmic domain of β-DG did not result in disruptions of the glia limitans while displaying a mild cerebellar migration defect [7]. This suggests that unlike β1Itg, whose intracellular signals are critical for cerebellar development, the extracellular interactions of α-DG are most crucial for cerebellar development.

In fact, the mucin domain of α-DG is decorated with LARGE-dependent phosphorylated O-mannosyl glycans that are responsible for α-DG high affinity binding activity [37,38]. The Large^myd^ mice harbor a spontaneous mutation the Large gene, which results in hypoglycosylated α-DG and loss of ligand binding [4,39]. These mice develop many of the brain malformations associated with severe forms of dystroglycanopathy, including disruption of the basement membrane and aberrant neuronal migration in the cerebrum and cerebellum [4,40,41]. A mouse model harboring the T192M mutation in DG shows impaired LARGE-mediated modification of phosphorylated O-mannosyl glycans on α-DG and reduced ligand-binding affinity [42]. However, these mice show no gross structural abnormality in the brain, suggesting that the residual ligand-binding activity of α-DG is sufficient for normal brain laminar development. Taken together, the pathological resemblance between the Large^myd^, nestin-Cre/DG-null, and GFAP-Cre/DG-null mice, and the lack of brain malformation in the T192M mice further support the hypothesis that extracellular interactions of α-DG in glia are critical for basal lamina integrity, a proper glial scaffold, and normal neuronal migration.

Although the distribution of GC ectopia was similar between GFAP-Cre/DG-null and nestin-Cre/DG-null mice, the severity of pathology was greater in nestin-Cre/DG-null cerebella. Since GFAP-Cre/DG-null mice retain expression of DG in PCs, it is possible that DG expression in PCs partially compensates for the loss of DG expression in other cell types or that PC DG has a function in GC migration. During their migration through the ML, GCs extend parallel fibers and make synaptic contacts with dendritic arbors of PC, and it has been suggested that adhesion molecules mediate the contacts between migrating neurons and PCs helping to direct migrating GCs into the IGL [43]. DG may be one of the adhesion molecules that mediates these interactions. However, genetic experiments using PCP2-Cre/DG-null mice revealed only small GC heterotopia compared to nestin-Cre or GFAP-Cre mice, showing that PC DG has a very minor role in cerebellar histogenesis. Alternatively, the mild phenotype observed in the PCP2-Cre/DG-null mice could be attributed to the small amount of residual DG expressed in PCs or the relatively late loss of DG during cerebellar development. While the postnatal inward radial migration of GC begins soon after birth and continues until P16, the PCP2 transgene is not expressed until P6 and does not become robust until P16 [23]. Similar experiments with malpha6-Cre/DG-null mice failed to discern a role for DG expression in GC. The malpha6 transgene is expressed in GC precursors during the peak of GC migration [24], yet no ectopic GC were observed in these mice.

Mapping the glia limitans and ectopic granule cell pathology in nestin-Cre and GFAP-Cre/DG null mice demonstrated a spatial heterogeneity along the glia limitans that has not previously been identified. Although our studies showed that perlecan, collagen IV, and laminin are all similarly perturbed at areas of disrupted basal lamina and all preserved at the surface of lobules IV-V and VI, we hypothesize that variation in spatial and temporal expression of extracellular matrix (ECM) proteins could explain the spatial heterogeneity of pathology observed in the nestin-Cre/DG null and GFAP-Cre/DG null mice. It is well established that expression of ECM components changes as basement membranes mature and likely involves the initial binding of laminin to dystroglycan with subsequent polymerization of further laminin chains and the cross-linking of the laminin superstructure with other ECM components [44]. Alternatively, there may be spatial and temporal differences in the glial endfeet receptors for ECM proteins during development, even though no spatial heterogeneity in the expression of dystroglycan or β1-integrin was observed in our work. Rather than viewing basement membranes within a given tissue as homogenous structures changing uniformly over time, further exploration of their assembly and maintenance as spatially heterogeneous structures is warranted.

## Conclusions

**Figure 12:**
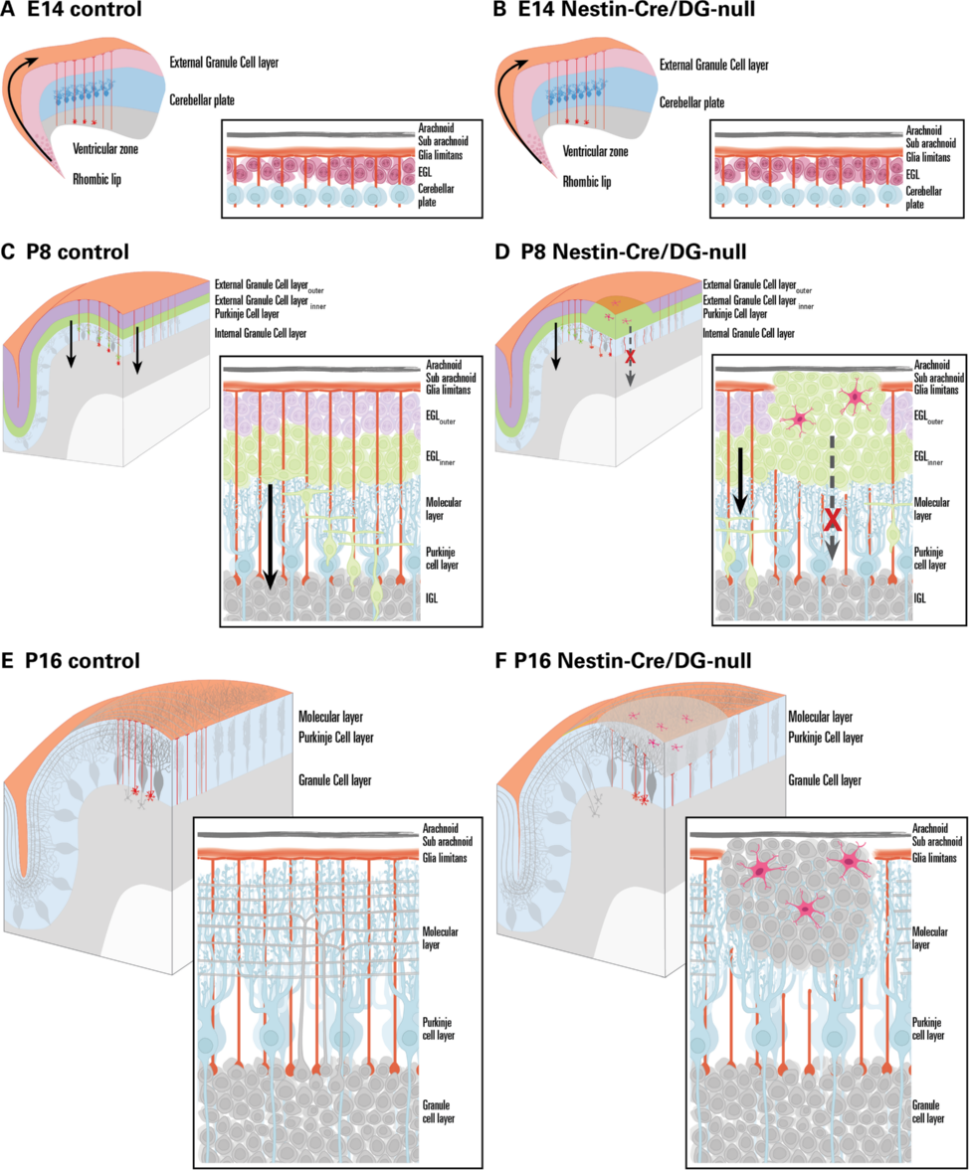
**Working model of abnormal cerebellar development in dystroglycanopathies.** In control mice at E14.5 **(A)**, the rhombic lip gives rise to granule cell precursors (GCPs), which migrate across the cerebellar surface to form a secondary zone of neurogenesis, the EGL. Radial glia from the vz extend their endfeet to the cerebellar surface, forming the glia limitans complete with basement membrane. In the nestin-Cre/DG-null mice **(B)**, DG is lost by E14.5 in all cells derived from the neuroepithelium; the cerebellar basement membrane at this time point remains intact. As cells continue to proliferate, the EGL is further divided into two zones: the outer EGL consisting of still-proliferating GCPs, and the inner EGL consisting of post mitotic GCs. GCs from the inner EGL will migrate inward, along radial glia, past the PC layer to settle in the IGL. The cerebellum at P8—the peak of post natal development—is illustrated in panel **C**. In the nestin-Cre/DG-null mice **(D)**, small breaks at the basement membrane are detectable on the day of birth then exacerbate. A failure of basement membrane assembly, basement membrane maintenance, and/or glial end foot-basement membrane adhesion, result in aberrant radial glia/Bergmann glia organization as the cerebellum grows. Post mitotic GCs from the inner EGL fail to migrate and remain trapped at the cerebellar surface due to lack of glial scaffolds or alterations in the local environment such as astrogliosis (red stellate cells). The EGL ceases to exist toward the end of normal post-natal development (P16, **E**). In the nestin-Cre/DG-null mice **(F)**, ectopic GCs are prominent at the cerebellar surface where the basement membrane is disrupted and Bergmann glia are highly unorganized. Despite their failure to migrate, these ectopic neurons express markers of differentiated GCs. Reactive gliosis is prominent within these regions of disrupted glia limitans and heterotopic GC.

In summary, we report that radial glia/Bergmann glia DG is essential for cerebellar development and propose a working model depicted in Figure 12. Dystroglycan, via its α-subunit, acts as an extracellular receptor for the ECM proteins that comprise the basement membrane (BM) covering the cerebellar surface to which the glial endfeet abut. Normal expression of matrix receptor proteins (i.e. DG and integrins) provide a platform for laminin binding and polymerization, as well as subsequent recruitment and cross-linking of other laminin-binding components to the self-assembled basal lamina. During the rapid expansion of cerebellar volume in the postnatal period, properly anchored glial endfeet provide not only a stabile platform on which BM components continue their assembly, but also maintain the radial scaffolding for neuronal migration. Insofar as GC progenitors from the EGL continue to proliferate, differentiate, and migrate radially inward over a protracted period of time, the integrity of the BM covering the expanding cerebellar cortex is critical for proper development. In the absence of functional α-DG, BM components may still be capable of assembling to a certain extent due to the presence of other cellular receptors (i.e. integrins). However, the lack of sufficient ligand-binding sites on the glial cell surface impairs BM stability. In fact, agrin and perlecan, both of which bind to laminin and α-DG, form collateral cross-linking bridges between the laminin and collagen networks, effectively compacting and stabilizing the BM. Loss of these interactions with α-DG appears sufficient to alter the biomechanical properties of the BM. The biomechanical stability of a different central nervous system basement membrane, the inner limiting membrane formed by Müller glial endfeet in the retina, was severely weakened in the Large^myd^ and POMGnT1-null mice [45]. We hypothesize that a similarly compromised BM on the surface of the cerebellum is likely to fracture during the rapid volumetric expansion of postnatal development, leading to reactive gliosis and the loss of Bergmann glial scaffolds. Collectively, this pathology is responsible for the granule cell migration abnormalities observed here and may be largely responsible for the cerebellar dysplasia observed in dystroglycanopathy patients.

## Competing interests

The authors declare that they have no competing interests.

## Authors’ contributions

HN planned and carried out experiments, interpreted data, and wrote the manuscript. APO carried out experiments, interpreted data, and drafted portions of the manuscript. JSS planned and carried out experiments, interpreted data, and drafted portions of the manuscript. SW processed tissue and performed histologic sectioning and staining. SERB assisted with genotyping and molecular verification of Cre expression. KPC planned experiments, interpreted data, and assisted with final edits of the manuscript. SAM planned and carried out experiments, interpreted data, and assisted with all aspects of writing the manuscript. All authors read and approved the final manuscript.

## Supplementary Material

Additional file 1: Figure S1 Regional heterogeneity in P8 cerebella. Lobules I, II, and III exhibit obliteration of their normal borders in nestin-Cre/DG-null (B) and, to a lesser extent, GFAP-Cre/DG-null (C) cerebella. The fissures bordering lobules IV and V showed extensive clusters of abnormally migrating granule cells in the nestin-Cre/DG-null cerebellum (E), while that of GFAP-Cre/DG-null (F) display subtle abnormalities. However, the surfaces of these lobules remained normal. In contrast, lobule IX displayed abnormal granule cells across its entirety in both nestin-Cre/ and GFAP-Cre/DG-null mice, with substantial fusion of between lobule VIII and IX. Asterisks (*) denote ectopic cells. Scale bar: 50 μm. Click here for file

Additional file 2: Figure S2 Fusion between lobules III and IV. H&E stainings of a control (A and A1) and nestin-Cre/DG-null cerebellum (B and B1) at post-natal day 21. Immunofluorescent staining of glial fibrilary acidic protein (GFAP, red) and laminin (LM, green) of the same control (C and C1) and nestin-Cre/DG-null cerebellum (D and D1). The basal lamina (green) was continuous between lobule III and IV in control cerebellum while Bergmann glial endfeet (red) abutted the glia limitans. In the nestin-Cre/DG-null cerebellum, a large break at the basal lamina (tildes; ~) was infiltrated by over extending Bergmann glial endfeet. Asterisks (*) indicate similar regions on all images. Scale bar: 20 μm. Click here for file

Additional file 3: Figure S3 Abnormal development of Purkinje cells. Immunostaining of calbindin (red) in adult control (A) nestin-Cre/DG-null cerebella (B and C). Purkinje cell somata (double daggers; ‡) formed a single layer above the GCL while their dendrites branched across the ML towards the cerebellar surface. However, PC dendrites in the nestin-Cre/DG-null appeared to reach across to adjacent lobules in areas of heterotopia and disrupted glia limitans. Daggers (†) denote abnormal PC dendrites. GCL = granule cell layer; ML = molecular layer; PCL = Purkinje cell layer. Scale bar: 20 μm. Click here for file

Additional file 4: Figure S4 Expression of sonic hedgehog (Shh) in the absence of dystroglycan. Immunofluorescent labeling of laminin or collagen IV (ColIV; red) and sonic hedgehog (Shh; green) in the developing cerebellum of wild-type (A-C, J) and nestin-Cre/DG-null (D-I, K, L) mice at P8 and P16. Shh expression in the cerebellum is not altered in the absence of dystroglycan. Carets (^) denote the intact basement membrane; tildes (~) indicate areas of disrupted basement membrane; asterisks (*) represent ectopic GCs. Scale bar = 50 μm. Click here for file

Additional file 5: Figure S5 Expression of AQP4 and Kir4.1. Immunodetection of collagen IV, aquaporin 4, and potassium inward rectifying channel 4.1 (ColIV, AQP4, and Kir4.1; all green) in control (A-C; G-I) and in nestin-Cre/DG-null cerebella (D-E; J-L) at P8 and P16. DAPI (blue) was used as nuclear counter stain. AQP4 is enriched at glial endfeet abutting the basement membrane (labeled with ColIV) in control cerebella; this concentration of AQP4 is reduced in the nestin-Cre/DG-null at P8 and lost by P16. Kir4.1 is expressed throughout the molecular layer of the cerebellum at P16; its expression is indistinguishable between nestin-Cre/DG-null and littermate control. Carets (^) denote the intact basement membrane; tildes (~) indicate areas of disrupted basement membrane; asterisks represent ectopic GCs. Scale bar = 50 μm. Click here for file

Additional file 6: Table S1 Area-specific incidence of pathology. Lobules IV and V displayed pathological breaks in the glia limitans rarely when compared to all other lobules, including IX. However, the fissures bordering IV and V were frequently abnormal. Click here for file
