# Supplementary figures and images for: Glial scaffold required for cerebellar granule cell migration is dependent on dystroglycan function as a receptor for basement membrane proteins
Source: Acta Neuropathol Commun. 2013 Sep 6;1:58. doi: 10.1186/2051-5960-1-58 (PMC3893534; doi:10.1186/2051-5960-1-58)

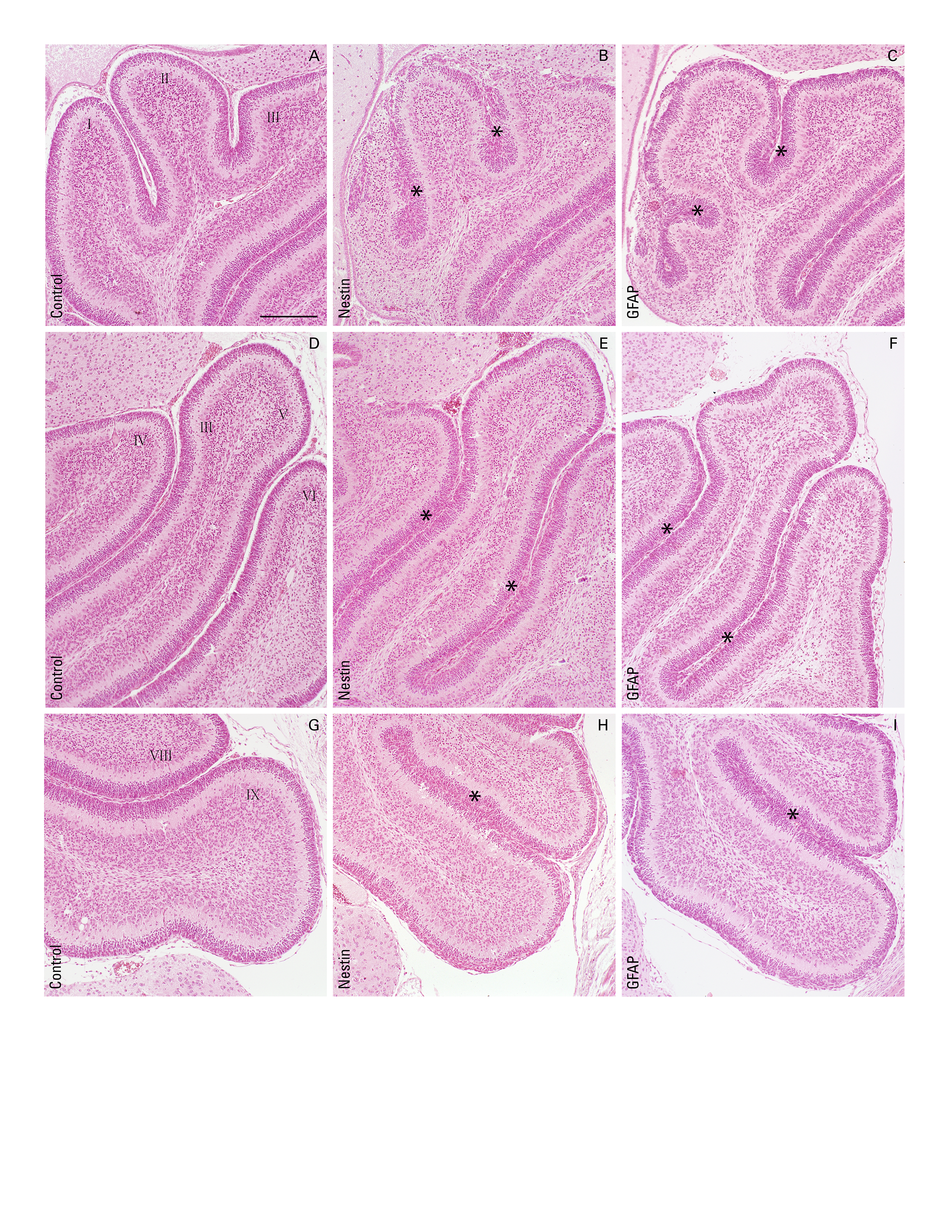

Supplement: Additional file 1: Figure S1 — Regional heterogeneity in P8 cerebella. Lobules I, II, and III exhibit obliteration of their normal borders in nestin-Cre/DG-null (B) and, to a lesser extent, GFAP-Cre/DG-null (C) cerebella. The fissures bordering lobules IV and V showed extensive clusters of abnormally migrating granule cells in the nestin-Cre/DG-null cerebellum (E), while that of GFAP-Cre/DG-null (F) display subtle abnormalities. However, the surfaces of these lobules remained normal. In contrast, lobule IX displayed abnormal granule cells across its entirety in both nestin-Cre/ and GFAP-Cre/DG-null mice, with substantial fusion of between lobule VIII and IX. Asterisks (*) denote ectopic cells. Scale bar: 50 μm. [file 2051-5960-1-58-S1.tiff]

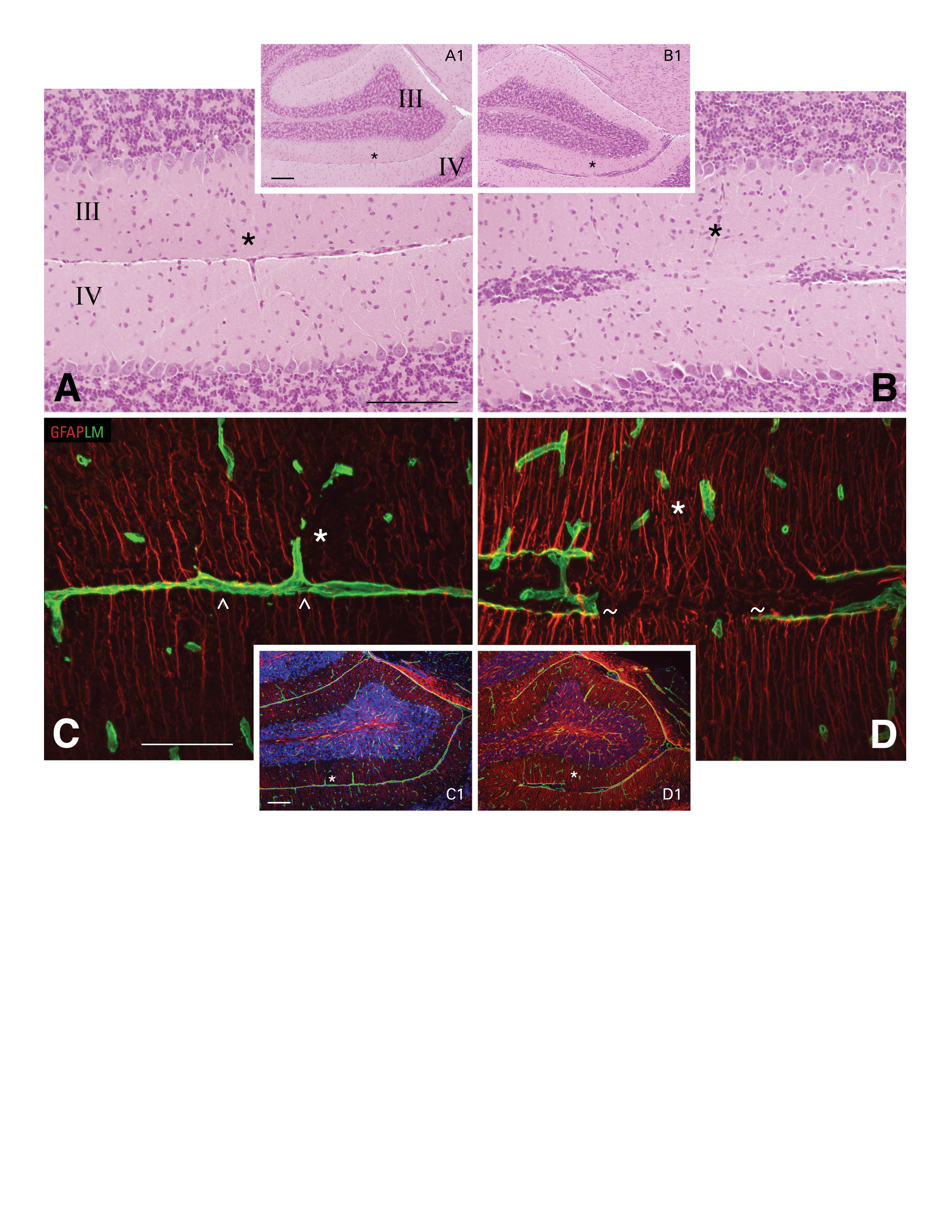

Supplement: Additional file 2: Figure S2 — Fusion between lobules III and IV. H&E stainings of a control (A and A1) and nestin-Cre/DG-null cerebellum (B and B1) at post-natal day 21. Immunofluorescent staining of glial fibrilary acidic protein (GFAP, red) and laminin (LM, green) of the same control (C and C1) and nestin-Cre/DG-null cerebellum (D and D1). The basal lamina (green) was continuous between lobule III and IV in control cerebellum while Bergmann glial endfeet (red) abutted the glia limitans. In the nestin-Cre/DG-null cerebellum, a large break at the basal lamina (tildes; ~) was infiltrated by over extending Bergmann glial endfeet. Asterisks (*) indicate similar regions on all images. Scale bar: 20 μm. [file 2051-5960-1-58-S2.tiff]

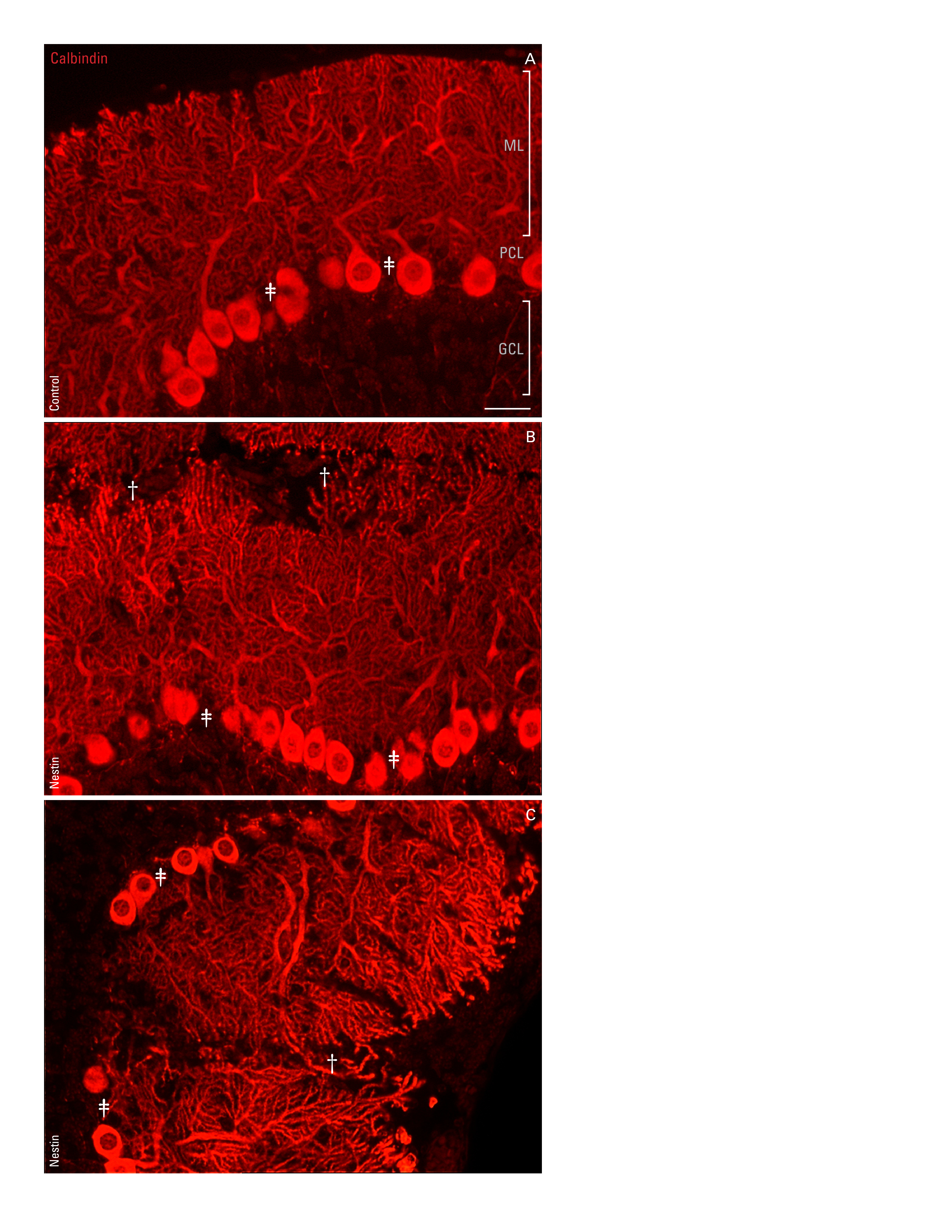

Supplement: Additional file 3: Figure S3 — Abnormal development of Purkinje cells. Immunostaining of calbindin (red) in adult control (A) nestin-Cre/DG-null cerebella (B and C). Purkinje cell somata (double daggers; ‡) formed a single layer above the GCL while their dendrites branched across the ML towards the cerebellar surface. However, PC dendrites in the nestin-Cre/DG-null appeared to reach across to adjacent lobules in areas of heterotopia and disrupted glia limitans. Daggers (†) denote abnormal PC dendrites. GCL = granule cell layer; ML = molecular layer; PCL = Purkinje cell layer. Scale bar: 20 μm. [file 2051-5960-1-58-S3.png]

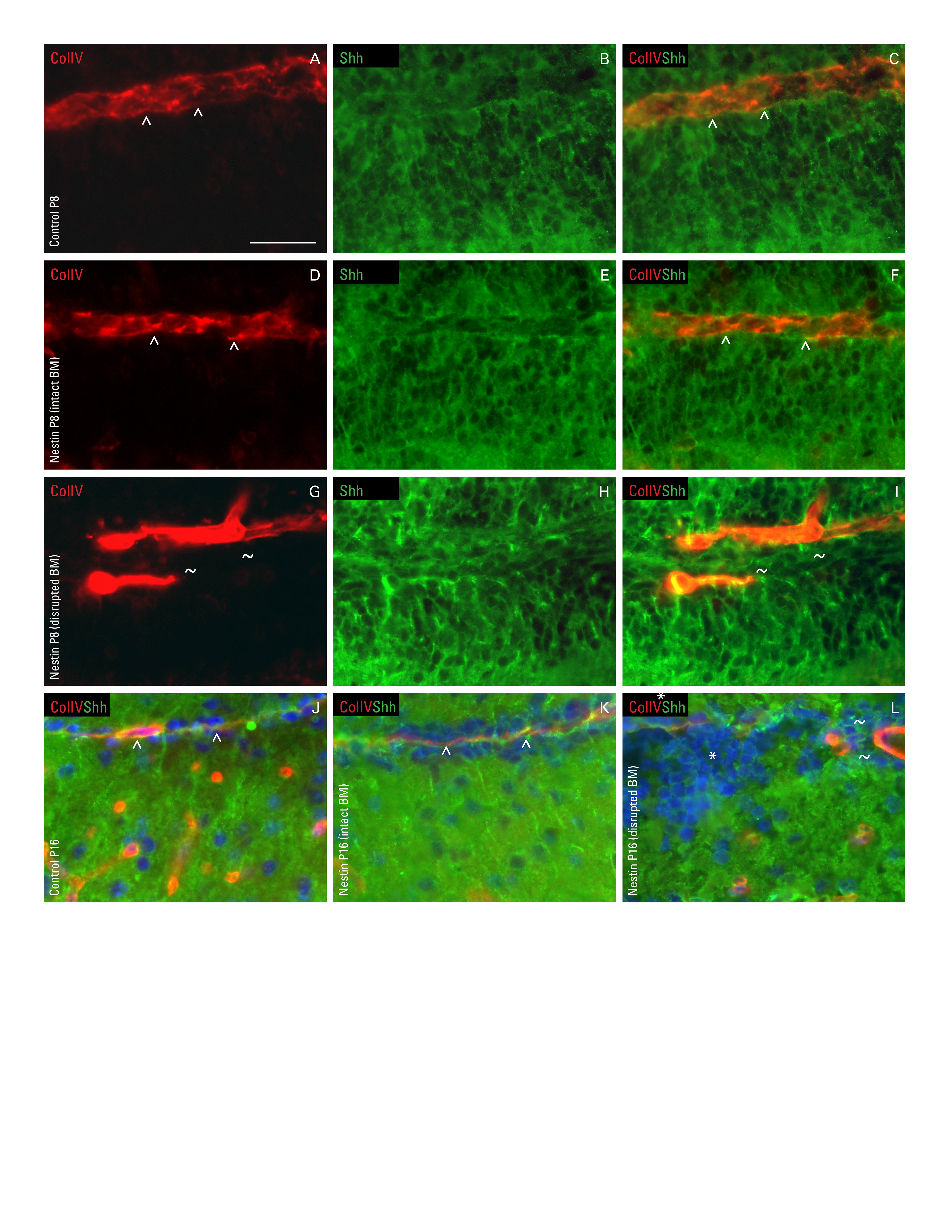

Supplement: Additional file 4: Figure S4 — Expression of sonic hedgehog (Shh) in the absence of dystroglycan. Immunofluorescent labeling of laminin or collagen IV (ColIV; red) and sonic hedgehog (Shh; green) in the developing cerebellum of wild-type (A-C, J) and nestin-Cre/DG-null (D-I, K, L) mice at P8 and P16. Shh expression in the cerebellum is not altered in the absence of dystroglycan. Carets (^) denote the intact basement membrane; tildes (~) indicate areas of disrupted basement membrane; asterisks (*) represent ectopic GCs. Scale bar = 50 μm. [file 2051-5960-1-58-S4.tiff]

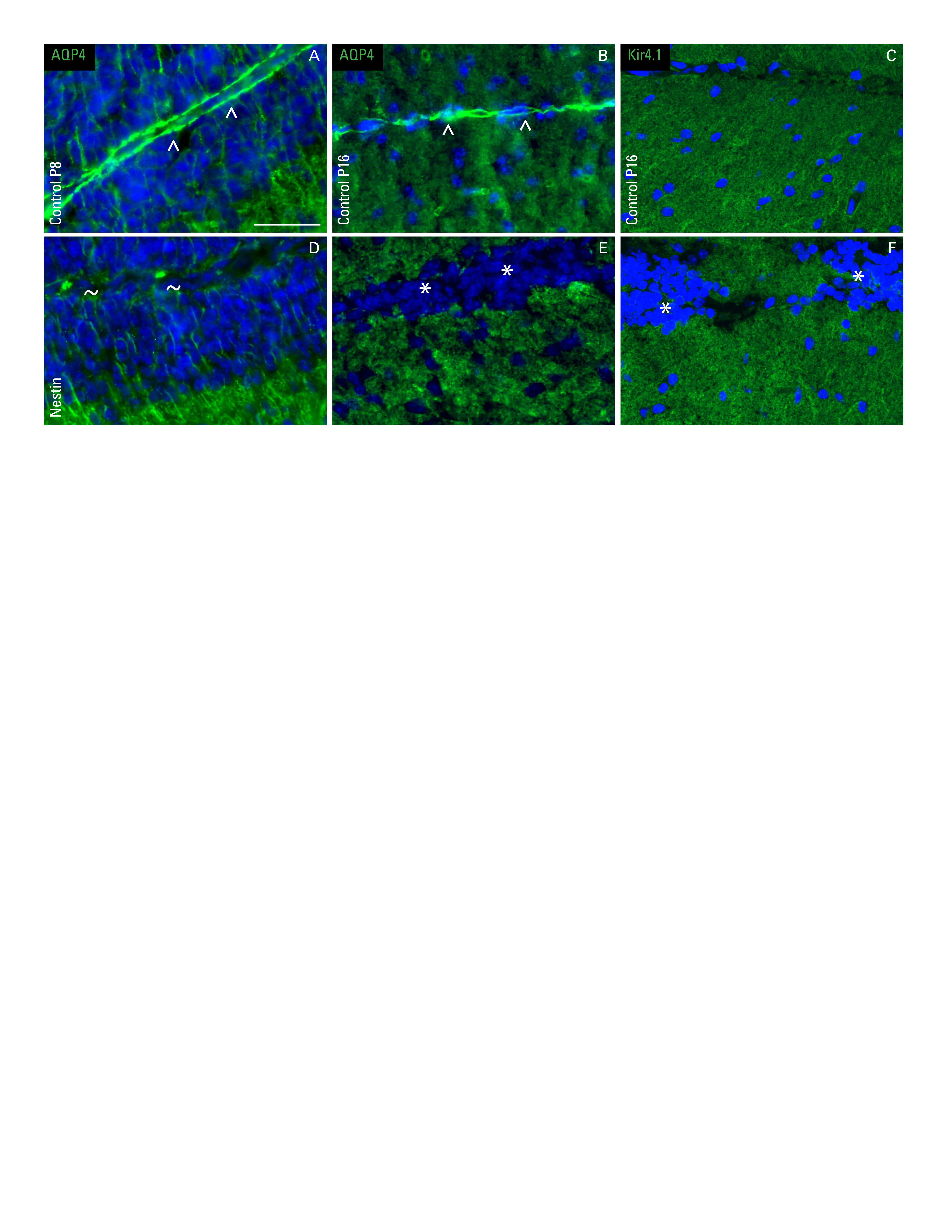

Supplement: Additional file 5: Figure S5 — Expression of AQP4 and Kir4.1. Immunodetection of collagen IV, aquaporin 4, and potassium inward rectifying channel 4.1 (ColIV, AQP4, and Kir4.1; all green) in control (A-C; G-I) and in nestin-Cre/DG-null cerebella (D-E; J-L) at P8 and P16. DAPI (blue) was used as nuclear counter stain. AQP4 is enriched at glial endfeet abutting the basement membrane (labeled with ColIV) in control cerebella; this concentration of AQP4 is reduced in the nestin-Cre/DG-null at P8 and lost by P16. Kir4.1 is expressed throughout the molecular layer of the cerebellum at P16; its expression is indistinguishable between nestin-Cre/DG-null and littermate control. Carets (^) denote the intact basement membrane; tildes (~) indicate areas of disrupted basement membrane; asterisks represent ectopic GCs. Scale bar = 50 μm. [file 2051-5960-1-58-S5.png]
