# Supplementary material for: Glial scaffold required for cerebellar granule cell migration is dependent on dystroglycan function as a receptor for basement membrane proteins
Source: Acta Neuropathol Commun. 2013 Sep 6;1:58. doi: 10.1186/2051-5960-1-58 (PMC3893534; doi:10.1186/2051-5960-1-58)
Supplement: Additional file 6: Table S1 — Area-specific incidence of pathology. Lobules IV and V displayed pathological breaks in the glia limitans rarely when compared to all other lobules, including IX. However, the fissures bordering IV and V were frequently abnormal. [file 2051-5960-1-58-S6.pdf]

	P2				P8				P16			
Lobule /Fissure	Nestin n=9		GFAP n=7		Nestin n=12		GFAP n=12		Nesin n=8		GFAP n=9	
I	7/9	78%	5/7	71%	12/12	100%	12/12	100%	8/8	100%	9/9	100%
II	4/9	44%	2/7	29%	11/12	92%	9/12	75%	7/8	88%	6/9	67%
III	2/9	22%	1/7	14%	9/12	75%	7/12	58%	6/8	75%	4/9	44%
F1	4/9	44%	1/7	14%	11/12	92%	11/12	92%	8/8	100%	9/9	100%
IV	0/9	0%	1/7	14%	0/12	0%	1/12	8%	0/8	0%	1/9	11%
V	0/9	0%	0/7	0%	0/12	0%	0/12	0%	2/8	25%	0/9	0%
F2	2/9	22%	1/7	14%	12/12	100	10/12	83%	7/8	88%	8/9	89%
VI	1/3	33%	1/7	14%	5/12	42%	3/12	25%	5/8	63%	6/9	67%
VII	2/9	22%	0/7	0%	8/12	67%	7/12	58%	7/8	85%	4/9	44%
VIII	4/9	44%	3/7	43%	8/12	67%	8/12	67%	6/8	75%	5/9	56%
F3	2/3	67%	2/7	29%	12/12	100%	12/12	100%	8/8	100%	9/9	100%
IX	8/9	89%	0/7	0%	12/12	100%	11/12	92%	7/8	88%	8/9	89%
F4	5/9	56%	4/7	57%	9/12	75%	9/12	75%	4/8	50%	8/9	89%
X	8/9	89%	4/7	57%	11/12	92%	9/12	75%	7/8	88%	8/9	89%

Supplemental Table 1. Area-specific incidence of pathology.
Lobules IV and V displayed pathological breaks in the glia limitans rarely when compared to all other lobules, including IX. However, the fissures bordering IV and V were frequently abnormal.
